# Enhancement of The Stability of Human Growth Hormone by Using Tris(hydroxymethyl)aminomethane: Molecular Docking and Experimental Analysis

**DOI:** 10.22074/cellj.2021.6903

**Published:** 2020-04-22

**Authors:** Siyavash Mirzaei, Hamid Mobedi, Hamid Gourabi, Mohammad Hosein Sanati, Sakine Khezli, Hamid Omidian, Masoume Ighaeie

**Affiliations:** 1. Department of Genetics, Reproductive Biomedicine Research Center, Royan Institute for Reproductive Biomedicine, ACECR, Tehran, Iran; 2. Department of Novel Drug Delivery Systems, Iran Polymer and Petrochemical Institute, Tehran, Iran; 3. Medical Genetics Department, National Institute of Genetic Engineering and Biotechnology (NIGEB), Tehran, Iran; 4. Department of Pharmaceutical Sciences, Nova Southeastern University, Pharmaceutical Sciences, Davie, Florida, USA; 5. Computational Nano Physical Chemistry Laboratory, Department of Chemistry, Azerbaijan Shahid Madani University, Tabriz, Iran

**Keywords:** Human Growth Hormone, Molecular Modeling, Protein Stability, Tris(hydroxymethyl)aminomethane

## Abstract

**Objective:**

It is so difficult to formulate human growth hormone (hGH) in a solution with high stability and new drug delivery system (NDDs) due to physiochemical instability. The purpose of this study was to investigate the possibility of using Tris as a hGH stabilizer.

**Materials and Methods:**

In this experimental study, the role of tris(hydroxymethyl)aminomethane (Tris) was evaluated as a hGH stabilizing agent in phosphate buffer, as a practical aqueous solution and a media to release NDDs. High- performance liquid chromatography (HPLC) and enzyme-linked immune sorbent assay (ELISA) were applied to investigate the stability of hGH in solutions and dynamic light scattering (DLS) was used to measure the effect of Tris on the hydrodynamic size of hGH in aqueous solutions. Ultra violet (UV) spectrophotometry was used to check the hGH spectrum. In computational study, formation of ligand-protein complex of the Tris-hGH, and the intermolecular interactions between Tris and hGH were studied by molecular docking modeling.

**Results:**

The results demonstrated that Tris at the optimum concentration, increases hGH stability in aqueous solutions. Also, molecular docking modeling confirmed that amino acid residues such as tyrosine (Tyr), proline (Pro), glutamic acid (Glu), aspartic acid (Asp), leucine (Leu), and phenylalanine (Phe) in hGH structure, were linked with Tris as a ligand.

**Conclusion:**

It seems that interactions between hGH and Tris are the most important reason for increment of the physicochemical stability of hGH in aqueous solutions containing Tris.

## Introduction

The protein of human growth hormone (hGH) has 191 amino acids and a molecular weight of 22 KDa. This protein is secreted and stored by somatotropic cells in the side section of the pituitary gland (1). Patients who suffer from GH deficiency, Prader-Willi, and Turner syndrome, receive a daily injection of this hormone (2). So, there is a strong need for new dosage forms that can facilitate hGH delivery, reduce the number of injections, and increase treatment efficacy and patients’ compliance (2-4). However, hGH instability in new drug delivery systems (NDDSs), aqueous solutions remain a major hurdle. So far, many studies have been done to increase the stability of hGH in solutions.

The most suitable stability for hGH in solution and NDDSs, is generally provided by the addition of antioxidants, osmolytes, acid neutralizers and biological buffers (5-9). In aqueous media, hGH stability is affected by buffer species, concentration, temperature, pH, ionic strength, and physical stress. These factors can produce unwanted crosslinking, oxidation, deamination, and consequently aggregation. An appropriate buffer for protein media, should preferably release its components from the protein domain (to enhance water surface tension), remove free radical, reduce the mobility of the water molecules, and suppress nucleophilic substitution on disulfide bonds (5, 10).

Biological buffers such as tris(hydroxymethyl) aminomethane (Tris) (C4H11NO3) and other tris derivatives are among additives used to stabilize proteins (11). Tris is a biocompatible weak base (molecular weight 121 Da) (12) that protects proteins against chemical degradation, denaturation, unfolding, and ultimately, aggregation by interacting with the protein, or similar to osmolytes, it induces proteins stabilization (10, 12).

In the previous studies, the stability of some proteins such as bovine serum albumin (BSA) and interleukin was increased in aqueous solutions at high temperatures in the presence of Tris (11, 13). It has become evident that the hydroxyl groups in Tris form hydrogen bond with glutamic acid, aspartic acid, alanine, glycine, tryptophan and cysteine amino acids in proteins and peptides, and protect them against chemical degradation (14). It was also shown that a greater hydrogen bonding can be achieved by increasing concentrations of Tris (11, 14). Changes in protein stability in the presence of Tris, at least in part, depend on intermolecular interactions that can be studied by empirical experiments as well as computer modeling. Intermolecular interactions that can be studied by empirical experiments as well as computer modeling. Molecular docking is a computer modeling approach used to predict the preferred orientation of binding to provide a stable conformation. The preferred orientation knowledge is utilized for prediction of the binding affinity between two molecules using scoring functions. Two approaches are commonly employed within the docking modeling association. One approach is to simulate the ligand and the protein as complementary surfaces. The second approach describes the docking process in which the energies of interaction in the ligand-protein pair are calculated (15). Investigation of the Tris effects on hGH stability and intermolecular interactions between hGH and Tris, was the aim of this study.

## Materials and Methods

### Materials

hGH was purchased in the form of powder with excipient ratio of 1:6 from GeneScience Pharmaceuticals (China). Tris(hydroxymethyl)aminomethane (Tris) was purchased from Sigma-Aldrich (USA), and n-propyl alcohol (analytical grade) was obtained from Merck, Germany.

### Sample preparation

In this experimental study, to compare hGH stability in phosphate and Tris buffers (0.05 M, pH=7.4), hGH solutions (1 mg/ml) were prepared and kept at two temperatures 5 ± 2℃ (refrigerator temperature) and 37 ± 1℃ (body temperature). The solutions were sampled and analyzed by High-performance liquid chromatography (HPLC) and enzyme-linked immune sorbent assay (ELISA), for 5 days and 24 hours, respectively. To investigate the effects of Tris on hGH stability in phosphate buffer, the hGH solutions (1 mg/ml) were prepared in phosphate buffer at the concentration of 0.05 M and pH=7.4, containing 0.0, 3.0, 4.0, and 6.0 mM of Tris and kept at 37 ± 1℃. The samples’ turbidity was tested after 48 hours. The hGH solutions (1 mg/ml) in Tris buffers (0.01, 0.03, 0.05, 0.07, 0.09 M and pH=7.4) and phosphate buffers (0.05 M, pH=7.4) containing of 0.00, 0.01, 0.03, 0.05, 0.07, 0.09 M Tris were prepared and kept at 37℃ for 24 hours. to investigate the effects of Tris on hGH hydrodynamic size, DLS test was performed.

To detect potential interactions between hGH and Tris, reference standard hGH solutions 1(mg/ml) containing 0.00, 0.05, and 0.1 M of Tris were prepared in distilled water and analyzed by UV spectrophotometry.

### Experimental analysis and evaluation

ELISA Kit: hGH concentration in solutions was measured using an ELISA Kit (Accubind, Monobind, USA). The absorbance was measured at the wavelength of 450 nm using a microplate photometer (Rodon, Titertek Multiskan, Netherlands).

HPLC: Analysis was performed according to the United State Pharmacopeia (USP) 40 by HPLC (smart line manager 5050, Knauer, Germany) with column C4 (4 mm×25 cm, 5 μm, 300 A°) (phenomenex, China), at 45℃ with mobile phase containing a mixture of 71% Tris buffer (0.05 M, pH=7.5) and 29% n-propyl alcohol and the flow rate was set at 0.5 ml/minute.

Dynamic light scattering (DLS): hGH aggregation and agglomeration was investigated using DLS (Omni, Brookhaven, USA) and turbidity tests.

UV spectrophotometer: hGH structural changes were tracked using a UV analyzer (UV-1650PC, Shimadzu, Japan).

### Computational analysis and evaluation methods

#### Density functional theory calculation

In the computational study, a full geometric optimization of the electronic ground state of Tris was obtained by applying the DFT (16, 17) using the Becke’s three-parameter hybrid exchange functional (B3) (18) and the Lee-Yang-Parr correlation functional (LYP) (19). A fairly large basis set with two sets of polarization functions denoted the 6-311G (2p, 2d) basis was used (16). All calculations were carried out using Gamess-US Package (20)

### Molecular docking

Computational docking can be used to predict bound conformation and free energies for a small ligand molecule binding a macromolecular target. Docking is used to study intermolecular interactions and their mechanisms (21).

The interaction of Tris, as a ligand, with hGH, as a macromolecule, was investigated. The Auto dock 4.2 was used to locate the appropriate conformations and binding orientations of one molecule of Tris into the hGH binding pocket. The lamarckian genetic algorithm (LGA) implemented in the Auto Dock program, was employed (22). The ligand (Tris) structure was optimized by DFT calculations. This optimized structure and hGH protein data bank [PDB code: 1Hgu (N-Hydroxyguanidine)] (23) were subjected to docking analysis. By adding the polar hydrogen atoms, the Kollman united atom charges, atomic solvation parameters, and fragmental volumes were assigned to the protein, using Auto Dock Tools (24). The grid spacing was 0.375Å, and each grid map consisted of 50×36×52 grid points. Lennard-Jones parameters 12- 6 and 12-10, supplied by the program, were used to model vander Waals interactions and hydrogen bonds, respectively. For ligand, random starting positions, random orientations, and torsions were used. The docking was performed with an initial 150 individuals’ population, a maximum of 270,000 generations, and maximum of 25 million energy evaluations. A maximum of 300 conformers was considered in the docking modeling process. Using a root mean square deviation (RMSD) less than 0.2 nm as a threshold, the resulting conformations were clustered. After the simulation was completed, the docked structure was analyzed and the interaction was investigated. The binding distance between the donors and acceptors and the hydrogen bond interactions were measured for the best conformers. Interactions, position, spacing of amino acids with each other within the complex of Tris and hGH, were also studied using a PyMOL package (25).

### Statistical analysis

In statistical analysis, to compare the data obtained from different samples, a t test (Paired Two Sample for Mean values) was performed using Microsoft Office Excel version 2016. A P<0.05 was considered statistically significant.

## Results

### Human growth hormone stability in the phosphate and Tris buffers

**Fig.1:**
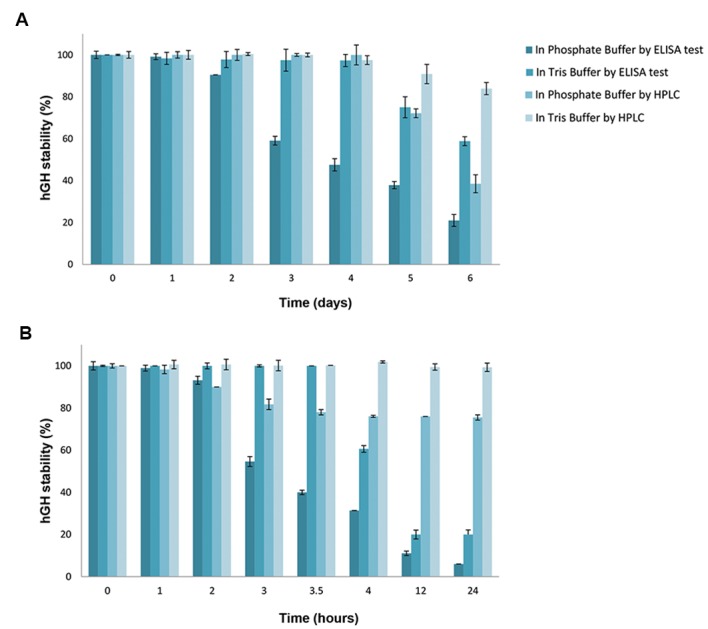
hGH stability in Tris and phosphate buffers (0.05 M, pH=7.4) analyzed by HPLC and ELISA tests. **A.** At 5 ± 2℃ and **B.** At 37 ± 1℃. hGH; Human growth hormone, HPLC; High performance liquid chromatography, and ELISA; Enzyme-linked immunosorbent assay

hGH chemical degradation such as oxidation and deamidation, and physical degradation (e.g. aggregation, agglomeration or unfolding), which lead to biological deactivation of hGH, in Tris and phosphate buffers (0.05 M, pH=7.4) at two temperatures of 5 ± 2˚C and 37 ± 1˚C, were investigated by HPLC (26, 27) and ELISA analysis (28), respectively (Fig.1).

HPLC analysis of hGH stability in phosphate buffer at 5 ± 2℃, showed that the stability of hGH in the phosphate buffer remained unchanged for four days; however, the results of ELISA analysis showed a decrease in the stability following the second day. In other words, hGH chemical degradation was found insignificant for four days, so decreased stability detected by ELISA test can be attributed to the physical degradation.

In Tris buffer, both HPLC and ELISA results confirmed that hGH stability remained unchanged for four days at 5 ± 2℃. Therefore, the difference in hGH stability between phosphate buffer and Tris buffer confirmed that Tris could enhance hGH stability twice as much. By comparison of the results of hGH stability study at 5 ± 2℃ in Tris and phosphate buffers, it was demonstrated that hGH stability remained unchanged for two days and then, significantly decreased in phosphate buffer (P≤0.035, Fig.1A).

The 24-hour physical and chemical stability data obtained by HPLC and ELISA analysis at 37 ± 1˚C, showed that hGH remained stable in phosphate buffer for 1 hour. hGH stability in this buffer decreased after one hour due to initiation of chemical degradations; however, after 2 hours, physical degradation also increased hGH instability as shown by ELISA results (Fig.1B).

The obtained HPLC and ELISA results in terms of hGH stability in Tris buffer showed that hGH chemical degradation was suppressed for 24 hours and hGH degradation after 3.5 hours, was related to physical degradation. Therefore, comparison of hGH stability between Tris buffer and phosphate buffer (37 ± 1˚C) in 3.5 hours, demonstrated a greater chemical stability for hGH in Tris buffer, and showed that hGH physical degradation rate in Tris buffer was lower than that in phosphate buffer (Fig.1B). Statistical analysis showed differences in hGH stability between Tris and phosphate buffer at 37 ± 1℃ from hour 2 to 24 (P=0.0209).

### Effect of Tris on human growth hormone stability in aqueous solution

**Fig.2:**
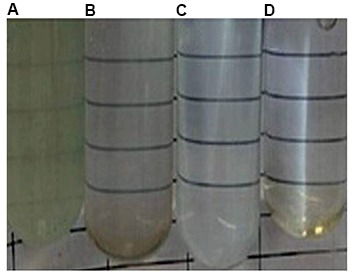
Human growth hormone (hGH) solution in phosphate buffer (pH=7.4), after 48 hours at 37 ± 1˚C. **A.** Solution containing 0.0 mM Tris, **B.** Solution containing 3.0 mM Tris, **C.** Solution containing 4.0 mM Tris, and **D. **Solution containing 6 mM Tris.

Since chemical and physical degradations can lead to protein aggregation, changes in the stability of a protein can be evaluated by monitoring protein aggregation. For this purpose, the effect of Tris on hGH stability was studied by adding Tris to phosphate buffer containing hGH. Aggregated hGH’s size and percent in phosphate buffer solution of hGH, was evaluated using qualitative turbidity and DLS tests performed at 37 ± 1℃. Following the addition of Tris, the color of the solution was changed from offwhite to completely transparent at Tris concentration of 3.0 up to 6.0 mM at 37 ± 1˚C after 48 hours. This color change was due to reductions in hGH physical degradation, such as unfolding, and aggregation, and chemical degradation (Fig.2).

**Fig.3:**
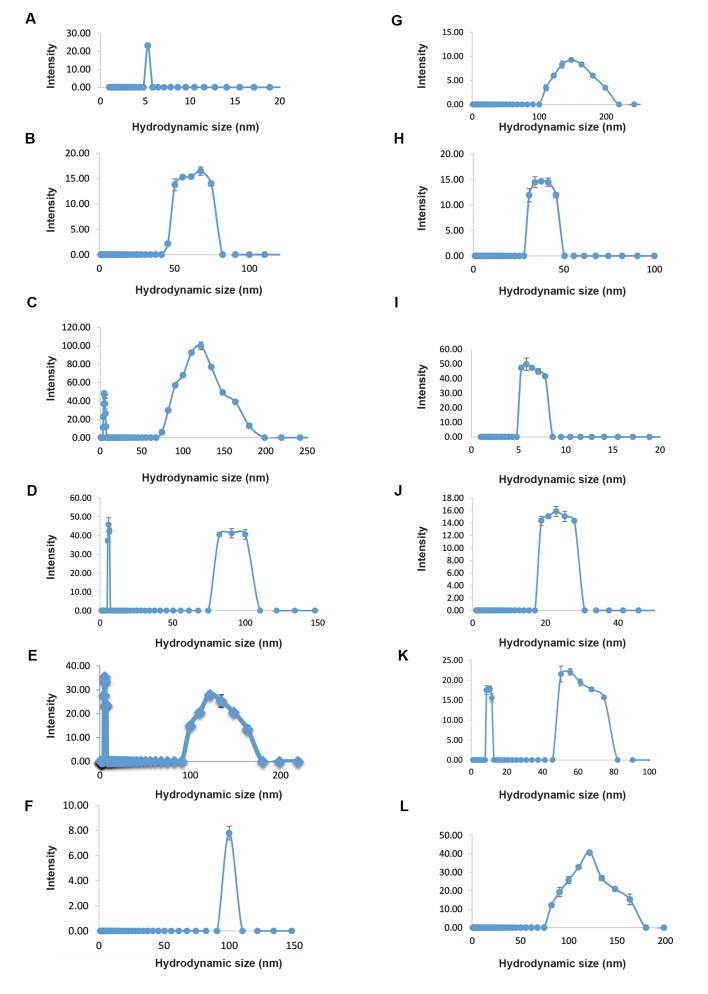
Particle size distribution of human growth hormone (hGH) protein in different phosphate and Tris buffer solutions, pH=7.4. **A.** At t=0, **B-F.** In Tris buffer 0.01, 0.03, 0.05, 0.07, and 0.09 M, **G-L.** In phosphate buffer containing 0.00, 0.01, 0.03, 0.05, 0.07, 0.09 M Tris after 24 hours at 37˚C.

The DLS test results indicated that adding Tris at the optimum concentration to the hGH solutions (Tris buffer and phosphate buffer containing Tris), increased the stability of this protein and prevented its aggregation (Fig.3). Based on these results, hydrodynamic size of hGH was in the range of 4 to 5 nm (in dimer form, hGH size is 8-10 nm) in both Tris and phosphate buffers (0.05 M, pH=7.4) at t=0 (Fig.3A). In Tris buffers (pH=7.4) of different concentrations, the average of aggregated protein diameter size decreased with increasing concentrations (0.01 to 0.05 M) of Tris from. Although hGH has the minimum average diameter size in Tris buffer 0.05 M, but with increasing concentration (from 0.05 to 0.09 M) of Tris, hGH aggregation and diameter size was increased (Fig.3B-F).

In phosphate buffer containing hGH and Tris, by increasing Tris concentration from 0 up to 0.03 M in solutions, the aggregated hGH diameter decreased to the diameter of the dimeric protein. However, by increasing Tris concentration from 0.03 up to 0.09 M, the aggregated protein diameter increased again (Fig.3G-L). Therefore, the minimum diameter of aggregated hGH was observed in Tris buffer 0.05 M, and phosphate buffer 0.05 M containing 0.03 M Tris, after 24 hours at 37˚C.

### Investigation of Tris and human growth hormone interaction

**Fig.4:**
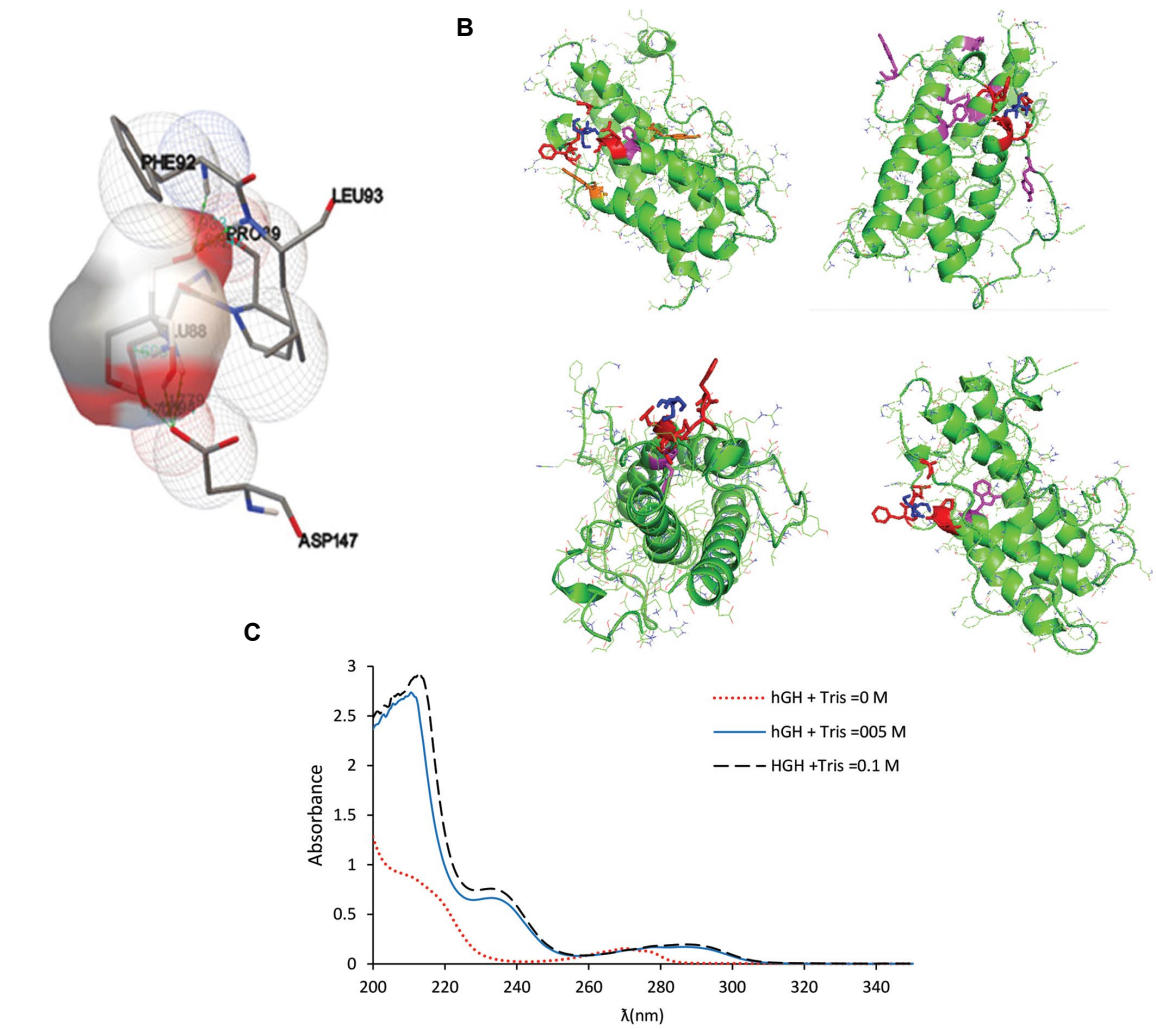
Interactions between human growth hormone (hGH) and Tris. **A.** Interaction from the close view (structure of the first conformation from the first cluster). **B.** Interactions between Tris (blue) and hGH amino acids involved in hydrogen bonds (red) and tryptophan (purple) at 3 different positions. **C.** Ultra violet (UV) spectrums related to hGH solutions containing 0, 0.05 and 0.1 M Tris.

Chemical interactions between hGH and Tris were investigated using UV spectrometry and molecular modeling. The preliminary results confirmed the interactions between the two molecules (Fig.4A-C). The amino acids of hGH that can potentially create hydrogen bonds with Tris, are introduced by Molecular Docking modeling in Table 1.

**Table 1:** Some results of Docking simulation


The cluster	The number of conformation in cluster	The lowest binding energy	The amino acids involved in hydrogen bonding

1	212	-6.22	PRO89, GLU88, ASP147, LEU93, PHE92
2	45	-5.86	GLU32, GLU29, TYR28, LYS41
3	3	-5.05	GLU118, LYS115, GLU119
4	28	-4.85	GLU33, GLu29, Glu32
5	3	-4.74	GLU119, GLU118
6	4	-4.57	GLU119, ASP118, Asp112
7	1	-4.46	ILE36, GLN40
8	1	-4.41	ASP109, ASP112, TYR111, VAL110
9	2	-4.09	TYR164, TYR28, LYS 41
10	1	-3.43	THR60, GLU65


**Fig.5:**
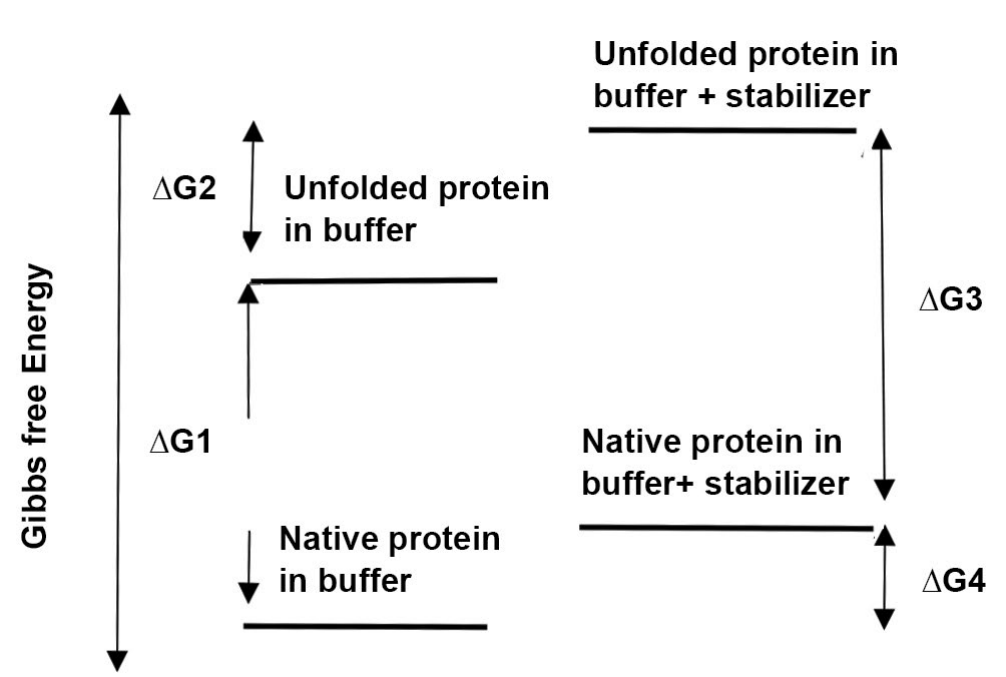
Transfer of natural protein to unfolded protein in buffer solution (∆G1); the reaction of unfolded protein in aqueous solution to the unfolded protein in buffer with stabilizer (∆G2); the transfer of natural protein to unfolded protein in buffer with stabilizer (∆G3); and the reaction of natural protein in aqueous solution to natural protein in solution with stabilizer (∆G4).

In addition to performed these tests by the present team, Studies by other researchers were suggested, Tris as an osmolyte can increase the hGH stability by changing in folded and unfolded hGH levels of Gibbs free energy (Fig.5).

## Discussion

With increasing concentrations of Tris, at lower concentrations (to 0.05 M in Tris buffer and to 0.03 M in phosphate buffer), the stability of hGH increased and at higher salt concentrations, increasing Tris concentrations led to decreasing hGH’s stability. Increases in hGH stability at low Tris concentration can be potentially attributed to the following three mechanisms: i. Interactions between Tris and hGH, ii. Salting-in effect of Tris, and/or iii. Preferential exclusion of Tris from protein domain in aqueous solutions.

Tris-hGH interactions: Tris could delay oxidation, deamidation, denaturation and ultimately, aggregation of hGH through electrostatic interactions and hydrogen bonding with hGH’s amino acids that are prone to degradation (10, 14).

Possibility of Tris-hGH electrostatic interactions can be explained by hGH and Tris net charge in solutions (29, 30). In solutions with pH=7.4, PI 5.12 and pKa 8, the protein could be negatively charged and Tris could be mainly ionized with the positive charges. Thus, probably, there were hGH-Tris electro-static interactions which could be regarded as the reasons for the increase in hGH stability in the solutions (31).

To investigate the possibility of hydrogen bonding between Tris and hGH, as a reason for improved stability of hGH molecules, the interactions between these two molecules were evaluated using a molecular modeling approach and spectrophotometric methods. Molecular docking is the most common means of analyzing ligandprotein interactions at the molecular level. Therefore, computational docking was used to obtain more details on the possible binding mode of Tris and Tris interaction with hGH residues.

Analysis of docking calculations using RMSD-tolerance of 2.0 Å out of 300 docking implementation, showed that there were 10 clusters of conformers. This included the number of conformations in every cluster, the binding energy of the lowest energy conformation in these clusters (in kcal/moll), and the residues participating in hydrogen bonds interactions. The lowest docked energy value was -6.22 kcal/moll, implying that Tris had affinity towards hGH. The residues participating in hydrogen bonds interactions, were Pro89, Glu88, Asp147, Leo93 and Phe92.

In addition to using molecular docking, UV experiment was also performed to study Tris- hGH interactions. UV technique is a simple method to study intermolecular interactions, structural changes and formation of molecular complexes (26, 32). The UV data confirmed that peaks of the hGH spectrum (tyrosine and tryptophan or phenylalanine) were shifted toward the longer wavelengths in the presence of Tris.

The interaction between tyrosin and phenylalanine with Tris, was proven in the Docking data; however, tryptophan could not directly interact with the ligand. The change in the UV peak of tryptophan in the presence of Tris, was presumably due to the interaction of the tryptophan amino acids with the neighboring amino acids in solutions. Consequently, molecular docking modeling and UV experiments data showed that the possibility of hydrogen bonding between Tris and the hGH.

Previous studies showed that some of the amino acids such as Asp, can cause hydrolytic degradation in hGH (33) or similarly, oxidation of Tyr and Leo can cause hGH degradation (34). Therefore, these electrostatic interactions and hydrogen bonds can prevent destructive chemical interactions.

Salting-in effect of Tris: Changes in hGH stability observed in different concentrations of Tris can be related to the Salting-in and Salting-out effects. In aqueous protein solutions, with increasing salt ions at low concentrations, the protein solubility increases due to the creation of charge-charge bonds between ions and the protein surface. This effect of salt is called salting-in. Salting-in effects are observed up to the optimum concentration, and then, with increasing ions concentration, protein solubility decreases (called salting-out). Salting-in phenomena in Tris buffer happened at concentrations lower than 0.05 M, and in phosphate buffer containing Tris, due to existence of phosphate ions, these phenomena happened at lower Tris concentration (0.03 M).

Increase in hGH’s physical stability, at low concentrations of Tris in solutions, is related to prevent protein aggregation (35).

Preferential exclusion from protein domain: Preferential exclusion of Tris from hGH’s domain in aqueous solutions, is the third mechanism underlying the increased hGH stability in solutions containing the optimum concentration of Tris like osmolyts molecules (36, 37).

Tris as an osmolyte can increase the hGH stability by changing in folded and unfolded hGH levels of Gibbs free energy (38).

The Gibbs free energy of native and unfolded protein in the aqueous solution containing an osmolyte, may be increased as much as ∆G4 and ∆G2, respectively. Since the ∆G2 is increasing more than ∆G4, the thermodynamic stability of the native protein is increased. In other words, unfolded protein in the solution containing a stabilizer is more unstable than in the solutions without any stabilizer (∆G3 is more than ∆G1). Therefore, the increased hGH’s stability could be mainly due to the decreased stability of the unfolded protein in the presence of Tris at the optimum concentration (38). In spite of increased hGH stability at low concentrations of Tris, its stability decreased at high concentration of this salt, from 0.05 to 0.09 in the Tris buffer and from 0.03 to 0.09 in the phosphate buffer. In these ionic strength, it seems that the net charge of protein was neutralized with near zero, and this increased the hydrophobic protein-protein interactions, leading to hGH aggregation. Also, salting-out effect at high salt concentrations in the protein solutions, leads to protein aggregation; in this phenomenon, salts ions could compete with the water molecules needed to solvate the proteins, that leads to protein aggregation (39).

Finally, it can be claimed that adding Tris to aqueous hGH solutions increases chemical and physical stability of this protein due to creation of Tris-hGH bonds, saltingin effect and Preferential exclusion of Tris, that prevents hGH aggregation. It can be also claimed that stabilization occurs at the optimum tris concentration, pH and appropriate ionic strength.

## Conclusion

The main finding of this study was that Tris as a weak base, increased the stability of hGH in aqueous solution at the body and refrigerator temperatures. The chemical degradation of hGH’s amino acids was decreased due to hydrogen bonding and electrostatic interactions with Tris in the proper pH and ionic strength. By changing the Gibbs free energy between the native and unfolded hGH, Tris prevented hGH from the aggregation. Therefore, as a stabilizer, Tris can enhance hGH physicochemical stability in solutions.
